# Synthetic LPETG-Containing Peptide Incorporation in the *Staphylococcus aureus* Cell-Wall in a Sortase A- and Growth Phase-Dependent Manner

**DOI:** 10.1371/journal.pone.0089260

**Published:** 2014-02-19

**Authors:** Silvie Hansenová Maňásková, Kamran Nazmi, Alex van Belkum, Floris J. Bikker, Willem J. B. van Wamel, Enno C. I. Veerman

**Affiliations:** 1 Department of Oral Biochemistry, Academic Centre for Dentistry Amsterdam, University of Amsterdam and VU University Amsterdam, Amsterdam, The Netherlands; 2 Department of Medical Microbiology and Infectious Diseases, Erasmus MC, Rotterdam, The Netherlands; Instituto Butantan, Brazil

## Abstract

The majority of *Staphylococcus aureus* virulence- and colonization-associated surface proteins contain a pentapeptide recognition motif (LPXTG). This motif can be recognized and cleaved by sortase A (SrtA) which is a membrane-bound transpeptidase. After cleavage these proteins are covalently incorporated into the peptidoglycan. Therefore, SrtA plays a key role in *S. aureus* virulence. We aimed to generate a substrate mimicking this SrtA recognition motif for several purposes: to incorporate this substrate into the *S. aureus* cell-wall in a SrtA-dependent manner, to characterize this incorporation and to determine the effect of substrate incorporation on the incorporation of native SrtA-dependent cell-surface-associated proteins. We synthesized substrate containing the specific LPXTG motif, LPETG. As a negative control we used a scrambled version of this substrate, EGTLP and a *S. aureus srtA* knockout strain. Both substrates contained a fluorescence label for detection by FACScan and fluorescence microscope. A spreading assay and a competitive Luminex assay were used to determine the effect of substrate treatment on native LPXTG containing proteins deposition in the bacterial cell-wall. We demonstrate a SrtA-dependent covalent incorporation of the LPETG-containing substrate in wild type *S. aureus* strains and several other Gram-positive bacterial species. LPETG-containing substrate incorporation in *S. aureus* was growth phase-dependent and peaked at the stationary phase. This incorporation negatively correlated with *srtA* mRNA expression. Exogenous addition of the artificial substrate did not result in a decreased expression of native SrtA substrates (e.g. clumping factor A/B and protein A) nor induced a *srtA* knockout phenotype.

## Introduction

*Staphylococcus aureus* is a Gram-positive pathogen that is the leading cause of skin, soft tissue, lower respiratory tract and bloodstream infections [1]. Its emerging multiple resistance to antibiotic treatment is becoming a major public health concern [2], [3]. Multiple resistant *S. aureus* strains, such as MRSA (methicillin resistant *S. aureus*), have become endemic in many parts of the world [4]. Vancomycin is the last therapeutic option available to treat *S. aureus* multi-drug resistant (MDR) infections. Therefore, the finding of the *vanA* gene, encoding vancomycin resistance, in *S. aureus* is worrisome [3]. So far, alternative therapeutic and preventive measures, such as anti-staphylococcal vaccines have not been successful [5]. The need for new therapeutics with different modes of action is considered urgent.

*S. aureus* expresses a broad spectrum of secreted and cell-surface-associated proteins during infection [1], [6]–[8]. These cell-surface-associated proteins are thought to play an important role during infections by facilitating adhesion to the host factors [9], [10]. The cell-surface-associated proteins are synthesized in the cytoplasm as precursors containing a unique N-terminal signal and a C-terminal sorting signal with a conserved recognition pentapeptide LPXTG motif [11], [12]. The N-terminus of the precursor peptide mediates secretion [13]. The peptide precursor can then be recognized by means of the LPXTG motif by sortase A (SrtA), a transpeptidase which catalyses the cleavage between the threonine and the glycine within the recognition motif [14]. The resulting free carboxyl group of threonine forms an amide bond with the free amino group of the pentaglycine within the peptidoglycan layer, leading to covalent anchoring into the staphylococcal cell-wall [15]. This cell-surface-associated protein anchoring can be inhibited by hydroxylamine [16]. This inhibition results in a release of these cell-surface-associated proteins into the medium.

Nelson *et al* [17] already previously synthesized a SrtA pseudo-substrate, containing an LPETG pentapeptide recognition motif. They showed a covalent incorporation into the cell-wall of *S. aureus* of this pseudo-substrate [17]. Based on their results, we generated a pseudo-substrate equipped with the LPETG motif as well. We aimed to characterize the incorporation of the pseudo-substrate into the cell-wall in a SrtA-dependent manner and to determine the effect of pseudo-substrate incorporation on the incorporation of native SrtA-dependent cell-surface-associated proteins. We found that the LPETG-containing substrate incorporation takes place in a growth phase dependent manner and peaks at the stationary growth phase. Furthermore, the treatment of *S. aureus* bacteria with the substrate alone did neither affect the deposition of the native LPXTG containing surface proteins nor induced the *srtA* KO phenotype.

## Materials and Methods

### Peptide Synthesis Procedure

FITC-labelled peptides were generated by solid-phase peptide synthesis using Fmoc-chemistry with a multi-peptide synthesiser (Syro II, MultiSynTech GmbH, Witten, Germany). Purification by RP-HPLC and confirmation of authenticity were performed as previously described [18]. Since the presence of a carboxylic acid group at the C-terminus of the sortase recognition motif (LPXTG) may inhibit SrtA activity, an amide group was incorporated at this position [19]. FITC-labelling was achieved by incorporating a fluorescein-containing lysine at the N-terminus of the LPXTG-amide. More detailed description of the peptide synthesis procedure and the chemical structure has been described elsewhere [17]. The substrates used in this study are shown in Table 1.

**Table 1:** Substrates used in this study.

Substrate name	Amino acids sequence
Native LPETG	K(FITC)LPETG-amide
Scrambled LPETG	K(FITC)EGTLP-amide

### Bacterial Strains

*S. aureus* 8325-4 wild type (WT) strain and its isogenic *srtA* deletion mutant (*srtA* KO) were used. This mutant was generated by *srtA:ermC* allele transduction with phage φ80α into *S. aureus* 8325-4 WT from *S. aureus* SH1000 *srtA* KO [20]. The 8325-4 *srtA* KO strain was selected on erythromycin plates (3 µg/ml). The absence of the intact *srtA* gene was confirmed with *ermC* and flanking *srtA* region PCR [20]. Beside 8325-4 WT we studied the Newman strain, a vancomycin intermediate *S. aureus* clinical isolate from Erasmus MC (VISA), a selection of bloodstream isolates obtained from Erasmus MC (B1, B4, B6 and B13), a selection of osteomyelitis isolates obtained from Erasmus MC (O1, O2, O3 and O4) and a selection of MRSA strains (N315, MU50, COL, BK2464, ANS46, HDE288, MW2, WIS, a pig isolate from a Dutch slaughterhouse, and a clinical MRSA isolate from Indonesia) [21]. Additionally, we studied a *sarA* (ALC 136) 8325-4 *S. aureus* KO strain, which was selected on erythromycin plates as well (3 µg/ml). Finally, we studied a selection of the Gram-positive clinical isolates: *Enterococcus faecium* (*E. faecium*) strains with none, VanA or VanB resistance pattern, *Streptococcus agalactiae* (*S. agalactiae*), *Staphylococcus cohnii* (*S. cohnii*) and *Enterococcus faecalis* (*E. faecalis*). The Gram-positive strains were all clinical isolates derived from the EU FP7 TEMPOtest-QC project (FP7-241742), except *S. cohnii*, which was an Erasmus MC bloodstream isolate.

### Growth Conditions

8325-4 WT and its isogenic *srtA* KO strain were grown overnight on respectively brain-heart infusion agar (BHA) (BD, Difco, Etten-Leur, the Netherlands) and BHA supplemented with 3 µg/ml erythromycin (Abbott Laboratories, U.S.). Subsequently, bacteria were re-suspended in Luria-Bertani (LB) (BD, Difco, Etten-Leur, the Netherlands) medium to obtain an optical density OD_600 nm_ of 0.1 and supplemented with either i) medium, ii) K(FITC)LPETG-amide or iii) a scrambled version of K(FITC)LPETG-amide: (K(FITC)EGTLP-amide). The final substrate concentration was 1 mM (unless otherwise indicated). The cultures were normalized to OD_600 nm_ 0.05 and cultured in a final volume of 50 µl in 96 wells-plates. The plates were incubated until either i) exponential (OD_600 nm_ 0.5), ii) post-logarithmic (OD_600 nm_ 1), iii) stationary (OD_600 nm_ 1.2) or iv) late stationary phase (24 hrs incubation) in the dark at 37°C upon continuous shaking (230 rpm). To study the substrate incorporation in Gram-positive isolates (other than *S. aureus*), the bacteria were re-suspended in Tryptic Soy Broth (TSB) (BD, BBL, Etten-Leur, the Netherlands) medium to obtain an optical density OD_600 nm_ of 0.1 and supplemented with either i) medium, ii) K(FITC)LPETG-amide or iii) a scrambled version of K(FITC)LPETG-amide: (K(FITC)EGTLP-amide) and incubated during 24 hrs.

To characterise the SrtA activity more in detail, 8325-4 WT, Newman, O1, B4, N315 and *srtA* KO *S. aureus* 8325-4 strains were incubated after normalization (OD_600 nm_ 0.05) in LB medium until the previously mentioned growth phases in the absence of substrate. Subsequently, bacterial densities were normalized to OD_600 nm_ 0.5 and after 3 PBS washes incubated with either i) SrtA buffer (50 mM Tris, 150 mM NaCl, 10 mM CaCl_2_, pH 7.5), ii) K(FITC)LPETG-amide in SrtA buffer or iii) a scrambled version of K(FITC)LPETG-amide in SrtA buffer in a final volume of 50 µl in 96 wells-plates during 1 hour in the dark at 37°C upon continuous shaking (230 rpm). The final substrate concentration was 1 mM.

To determine the effect of the hydroxylamine on the substrate incorporation, 8325-4 WT and *srtA* KO *S. aureus* 8325-4 were grown to stationary phase, washed 3 times with PBS, normalized (OD_600 nm_ 0.5) and incubated either with SrtA buffer or substrate dissolved in SrtA buffer as mentioned above. Additionally, serial dilutions (0, 5, 10, 50 and 100 mM) of NH_2_OH (hydroxylamine hydrochloride, Sigma-Aldrich Zwijndrecht, the Netherlands) were added and the plate was incubated overnight in the dark at 37°C upon continuous shaking (230 rpm).

The bacteria were washed four times with PBS followed by centrifugation during 5 min at 3,700× *g*. To remove the non-covalently bound and intracellular substrate, bacteria were washed with 1% of sodium dodecyl sulphate (SDS) (Merck-Schuchardt OHG, Germany) at 60°C during 5 min. Subsequently, bacteria were washed again with PBS four times followed by centrifugation for 5 min at 3,700× *g*. Pelleted bacteria were re-suspended and used for microscopy and flow-cytometry.

### Fluorescence Microscopy and Flow-cytometry

Labelled bacteria were re-suspended in PBS and analysed with a phase contrast fluorescence microscope (Olympus IX51, Leiden, the Netherlands). For the flow-cytometry, labelled bacteria were re-suspended in 100 µl of 4% formaldehyde (Sigma-Aldrich, Zwijndrecht, the Netherlands) in PBS and incubated for 45 min at ambient temperature under continuous agitation. After 3 washes with PBS, bacteria were re-suspended in 0.5 ml PBS. Stained bacteria were acquired on a FacsCanto II™ flow cytometer and analysed using FacsDiva™ software (BD Biosciences). All FACScan analysis were performed as three independent experiments. The SEM was calculated from these triplicates.

### RNA Isolation

Two representative strains (O1 and 8325-4 WT) were used for RNA isolation. Bacteria were cultured until exponential, post-logarithmic, stationary and late stationary phase (based on OD measurement) in LB medium as previously described. Subsequently, 10 ml of each bacterial culture was centrifuged at 3,700× *g* during 5 min at 4°C. The pellet was re-suspended in 3.3 ml RNA protect™ Bacterial Reagents (Qiagen, Germantown, MD, USA) and left to stabilize for 5 min at ambient temperature. The bacteria were collected by centrifugation at 3,700× *g* during 10 min at ambient temperature. The pellet was re-suspended in 1 ml RNA-pro solution (Fast RNA Pro Blue kit from MP Biomedicals, Qbiogene Inc., City, CA, USA) and stored at −20°C until use. RNA was isolated according the manufacturer’s protocol (Fast RNA Pro Blue kit from MP Biomedicals).

### Reverse Transcription and Quantitative Real Time-PCR

Samples were treated with DNAase to remove the DNA contamination from the RNA samples. We added 6 µl 10x Turbo DNase buffer (Ambion, Austin, TX, USA) together with 2 U Turbo DNAse (Ambion, Austin, TX, USA) to 10 µg of RNA in DEPC-treated water (Ambion, Austin, TX, USA) and incubated 30 min at 37°C in a final volume of 60 µl in DEPC-treated water. After 30 min of incubation, 2 U of new Turbo DNase was added and incubation proceeded for another 30 min at 37°C. The reaction was stopped by addition of 0.2 volumes of DNAse inactivation reagent (Ambion, Austin, TX, USA) during 2 min at ambient temperature and with occasional mixing. RNA containing supernatants were collected by centrifugation during 1.5 min at 9,000× *g* at ambient temperature. To remove all DNA remnants, 2 µg of each Ambion DNase treated RNA samples were incubated 30 min at 37°C with 2 U of DNase (Fermentas, St. Leon-Rot, Germany) with 2 µl of reaction buffer with MgCl_2_ in a final volume of 20 µl (in DEPC-treated water). The reaction was stopped by addition of 2 µl of 25 mM EDTA (Fermentas, St. Leon-Rot, Germany) and 10 min incubation at 65°C followed. 1 µg of total mRNA was reverse transcribed into cDNA using 200 U RevertAid H Minus Reverse transcriptase (Fermentas, St. Leon-Rot, Germany), 4 µl of 5x reaction buffer (Fermentas, St. Leon-Rot, Germany), 1 µl of random hexamer primers (Fermentas, St. Leon-Rot, Germany), 20 U of RiboLock RNase inhibitor (Fermentas, St. Leon-Rot, Germany) and 2 µl of 10 mM dNTP mix (Fermentas, St. Leon-Rot, Germany) in a final volume of 20 µl. The reaction was performed according to the instructions of the Reverse transcriptase manufacturer (Fermentas, St. Leon-Rot, Germany). cDNA samples were diluted 1∶10 and stored at −20°C until use.

Quantitative real time PCR was carried out using the Light Cycler instrument (Roche, Woerden, the Netherlands) in combination with the Light Cycler DNA amplification kit SYBR Green I (Roche, Woerden, the Netherlands). Master mixes were prepared according to the manufacturer’s instructions using target-specific primers (listed in table 2). We performed the amplification reaction at conditions described earlier with a modified annealing temperature of 58°C [22]. The *srtA* mRNA expression was normalized to the *gyrB* mRNA expression and quantified by relative expression assays (delta/delta CT method). The specificity of the PCR was verified by ethidium bromide staining of 2% agarose gels after gel electrophoresis of the reaction product.

**Table 2:** Oligonucleotide primers used in this study [39].

Targetgene	GenBankaccession no.	Primer	Primer sequence, 3′→5′
*gyrB*	D10489	GyrB F	TTAGTGTGGGAAATTGTCG
		GyrB R	AGTCTTGTGACAATGCGTT
*srtA*	AF162687	SrtA F	CGATTAATGACAATCGCT
		SrtA R	TAATTGTTCAGGTGTTGCTG

### *S. aureus* Fibrinogen and Fc-region Binding

*S. aureus* 8325-4 WT and *srtA* KO strains were grown until exponential growth phase (OD_600 nm_ 0.5) in LB and Brain Heart Infusion (BHI) (BD, Difco, Etten-Leur, the Netherlands) media in the presence of either K(FITC)LPETG-amide or K(FITC)EGTLP-amide substrates. After 3 PBS washes, bacteria were incubated with fluorescently labelled fibrinogen (DyLight 633 NHS Esther, Thermo Scientific, Landsmeer, the Netherlands) or with a 1/100 dilution of fluorescently labelled (DyLight 633 NHS Esther, Thermo Scientific, Landsmeer, the Netherlands) human IgG Fc protein (Fitzgerald Industries International, Bio-Connect BV, Huissen, the Netherlands) in a volume of 10 µl, during 1 hr and upon continuous agitation at ambient temperature in the dark. Fibrinogen and IgG Fc protein labelling was performed according to the manufacturer’s protocol. The bacteria were washed 3 times with PBS and their fluorescence was determined on a FacsCanto II™ flow cytometer and analysed using FacsDiva™ software (BD Biosciences).

### Semi-quantification of *S. aureus* Cell-surface-associated Proteins Upon Substrate Incubation by a Competitive Luminex

8325-4 WT and *srtA S. aureus* KO strains OD_600 nm_ 0.05 were cultured until exponential growth phase in BHI medium upon addition of either i) medium, ii) K(FITC)LPETG-amide or iii) K(FITC)EGTLP-amide (final volume of 200 µl), the final substrate concentration was 1 mM. The cultures were centrifuged at 3,700× *g* at 4°C during 5 min. The bacterial pellet was additionally washed three times with PBS. After the last wash-step, the bacterial pellet was re-suspended in 140 µl assay buffer (PBS, 1% bovine serum albumin, 0.5% sodium azide, pH 7.4) and tenfold serial dilution were made. To determine the effect of substrate treatment on the covalent binding of native cell-surface-associated proteins, other series of bacterial pellets were treated with 1% SDS in PBS (Merck-Schuchardt) at 60°C during 5 min. The SDS-treated pellet was washed three times with PBS and tenfold serial dilutions were made as well. 100 µl of the bacterial pellet dilutions were separately incubated with 100 µl 1/100 diluted HPS (human pooled serum) upon continuous shaking 800 rpm, at 4°C during 35 min (thermomixer plate shaker, Eppendorf). The samples were centrifuged at 2,400× *g* during 10 min after the incubation with HPS. 80 µl of the bacterial cell-pellet-derived supernatant was carefully collected. These supernatants were centrifuged at 2,400× *g* during 15 min. 60 µl of second supernatants were stored at 4°C until use. The levels of the non-captured *S. aureus* cell-surface-associated protein antibodies were simultaneously quantified in the supernatants using Luminex bead based flow cytometry (Luminex Corporation). Antigens used in this assay were clumping factors A/B (ClfA and ClfB), iron-regulated surface determinant A (IsdA), *S. aureus* alpha-toxin (α-tox), *S. pneumoniae* choline-binding protein A (CbpA) and human Metapneumovirus (hMPV-99). All antigens were coupled to SeroMAP carboxylated beads (Luminex Corporation) as described previously [23], [24]. The final concentration of the beads was adjusted to 3,000 beads/µl and the beads were stored until use at 4°C in the dark. α-tox (not expressed during the exponential growth phase) and the non-*S. aureus* proteins (CbpA and hMPV-99) were used as negative controls. More detailed description of the competitive Luminex assay are described elsewhere [25].

### Colony Spreading Assay

A colony spreading assay was performed as previously described by Kaito *et al* and Tsompanidou *et al*, who showed a hyper-spreading phenotype of *srtA* KO strain [26], [27]. In brief, Tryptic Soy Broth (TSB) (BD, BBL, Etten-Leur, the Netherlands) was supplemented with 0.24% Agar powder (BD, Difco, Etten-Leur, the Netherlands) to prepare TSA soft agar. Fifteen ml TSA soft agar was poured into plates with 8 cm diameter and dried for 20 min. In parallel, SH1000 WT and isogenic *srtA S. aureus* KO strains OD_600 nm_ 0.05 were cultured during 24 hrs in TSB medium upon addition of either i) medium, ii) K(FITC)LPETG-amide or iii) K(FITC)EGTLP-amide (final volume of 50 µl), the final substrate concentration was 2 mM, 1 mM and 0.5 mM. After 24 hrs incubation 2 µl of individual cultures were gently spotted on the TSA soft agar plates and dried for 15 min. The plates were incubated during 20 hrs at 37°C. After incubation the spreading zones were examined and pictures were taken.

### Statistical Methods

Statistical analysis was performed with the Prism 5.0 package (GraphPad software, San Diego, CA, USA). We used non-parametric Mann Whitney testing, one-way ANOVA testing with Bonferroni correction and Pearson correlation testing, considering P<0.05 as being statistically significant in all cases.

## Results

### An Exogenous Native LPETG Substrate is in a SrtA-dependent Manner Covalently Incorporated Into the Cell-wall of *S. aureus* WT Strains

**Figure 1:**
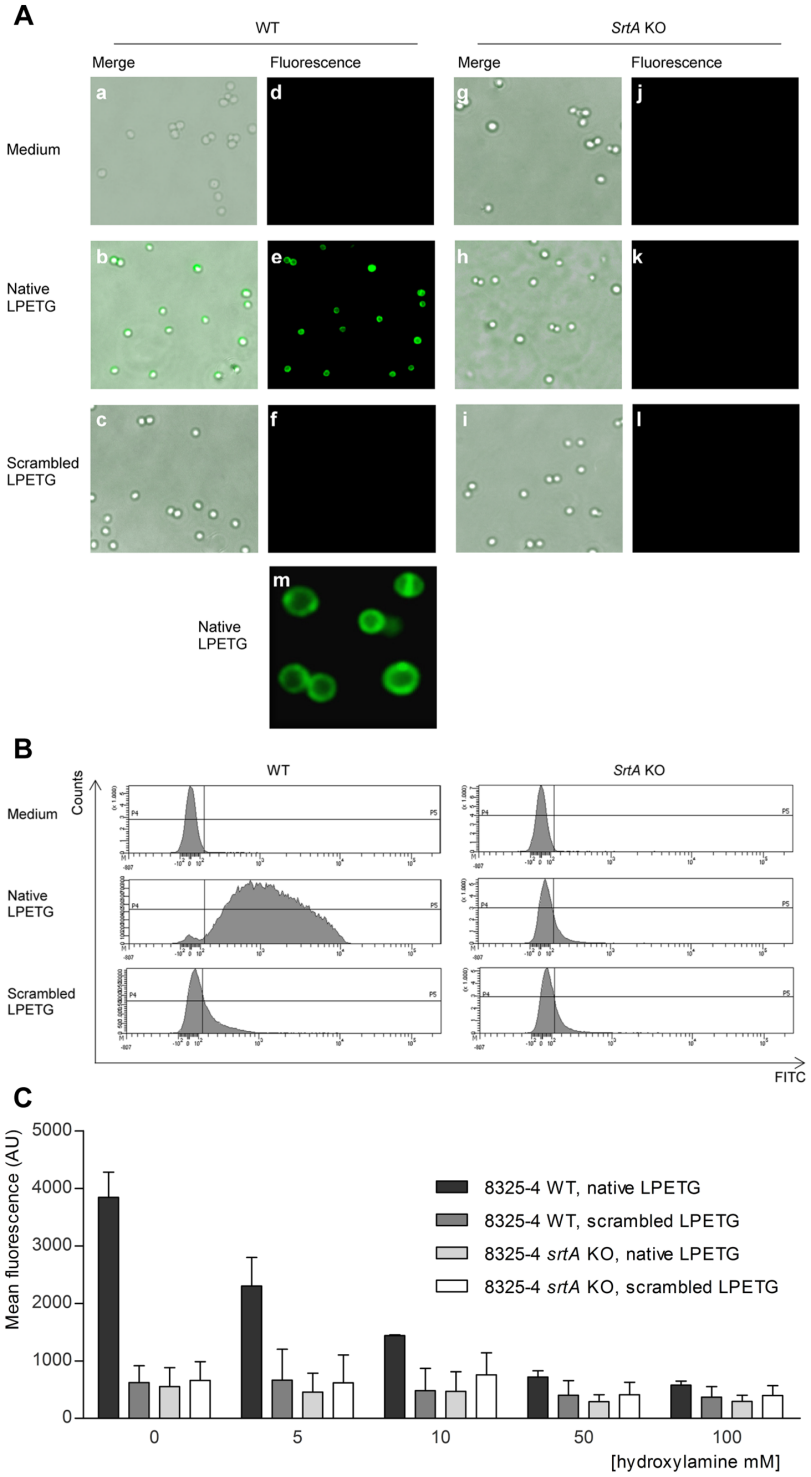
Native LPETG substrate is incorporated in large quantities in a SrtA-dependent manner into the *S. aureus* 8325-4 WT cell-wall and incorporation can be inhibited by hydroxylamine. The *S. aureus* 8325-4 WT and *srtA* KO strains were cultured in the presence of 1 mM native or scrambled LPETG substrates in LB medium during 24 hrs. Bacteria were washed, re-suspended in PBS and analysed with (A) fluorescence microscopy and (B) FACS. Abbreviations: WT: wild type *S. aureus* (8325-4), *srtA* KO (isogenic *srtA* deletion mutant), merge: fluorescent images superimposed on top of brightfield images, fluorescence: fluorescence images, medium: bacteria incubated only in LB medium, native LPETG: bacteria incubated with 1 mM K(FITC)LPETG-amide substrate diluted in LB medium, scrambled LPETG: bacteria incubated with 1 mM K(FITC)EGTLP-amide substrate diluted in LB medium. The microscopy images are a representation of at least 5 independent experiments. The histogram of FACS data represents the FITC fluorescence intensity on the x-axis and the bacterial count on the y-axis. FACS scan analysis is a representation of 3 independent experiments. (C) Effect of hydroxylamine on native LPETG incorporation. Bacteria were grown until stationary growth phase was reached, washed, normalized to OD_600 nm_ 0.5 and incubated overnight with 1 mM native or scrambled LPETG substrates in SrtA buffer upon addition of 0, 5, 10, 50 or 100 mM of NH_2_OH. After incubation, bacteria were analysed by FACS. The mean fluorescence intensity, reflecting the quantity of the incorporated substrate per bacteria, was plotted on the y-axis. This is a representative image of independently performed FACS analysis in triplicate with the SEM depicted.

To study the substrate incorporation, we incubated *S. aureus* bacteria with either K(FITC)LPETG-amide (native LPETG) or K(FITC)EGTLP-amide (scrambled LPETG) in LB medium until the late stationary phase. After the incubation, we treated the bacteria with 1% SDS to remove non-covalently (a-specifically) bound and internalized substrate. The microscopic analysis of the 8325-4 WT strain cultured with native LPETG substrate displayed strong cell-wall-associated fluorescence (Fig. 1b and e). Neither control experiments with medium alone (Fig. 1a and d), nor with the scrambled version of the peptide showed detectable cellular fluorescence (Fig. 1c and f). Furthermore, the *srtA* KO strain displayed virtually no fluorescence when cultured in the presence of native LPETG substrate (Fig. 1 h and k). The fluorescence data therefore demonstrate that the SrtA specific substrate is incorporated into the cell-wall of *S. aureus* WT strain in a SrtA-dependent manner. Culturing of bacteria in the presence of these sortase substrates did not affect bacterial growth and viability (data not shown). Finally, the incorporated native LPETG substrate localizes predominantly to the bacterial perimeter in the *S. aureus* cell-wall (Fig. 1 m).

To quantify the substrate incorporation, we analysed 8325-4 WT and the KO bacteria with the FACScan (Fig. 1B). WT bacteria incubated with native LPETG substrate showed high fluorescence, whereas *srt*A KO bacteria showed no significant increase in fluorescence. Incubation with the scrambled substrate did result in increased fluorescence in neither of the two strains (Fig. 1B). To further explore the role of SrtA, incorporation of external sortase substrate was tested in the presence of hydroxylamine, which acts as a soluble acceptor for SrtA [16]. In the presence of hydroxylamine, a dose-dependent inhibition of the native LPETG substrate incorporation into the cell-wall of *S. aureus* 8325-4 WT strain occurred (Fig. 1C). These data all together conclusively show that the exogenous native LPETG substrate is efficiently processed by the endogenous *S. aureus* SrtA.

**Figure 2:**
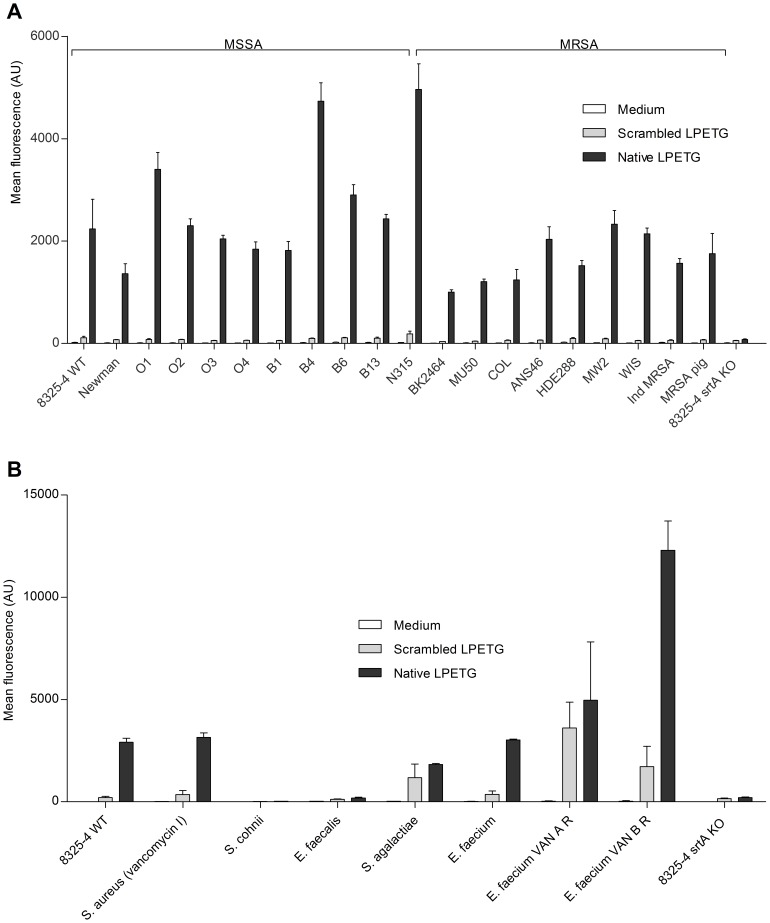
Several *S. aureus* and other Gram-positive strains incorporate different levels of native LPETG substrate after 24 hrs of incubation. (A) FACS analysis of 10 MSSA and 10 MRSA *S. aureus* isolates. All *S. aureus* strains were incubated in LB medium as described in figure 1A. (B) FACS analysis of a selection of Gram positive bacteria. The bacteria were cultured in the presence of 1 mM native or scrambled LPETG substrates during 24 hrs in TSB medium. Bacteria were washed, re-suspended in PBS and analysed with FACS. Bacterial strains used are depicted on the x-axis. Abbreviations used are described in figure 1. These are the representative images of independently performed FACS analysis in triplicate with the SEM depicted.

To determine if strains other than *S. aureus* 8325-4 WT can incorporate the native LPETG substrate, 10 MSSA and 10 MRSA strains were analysed. All tested *S. aureus* strains showed only incorporation of the native LPETG substrate (Fig. 2A). Strain-specific differences in incorporation rates were observed. As SrtA transpeptidase is widely distributed among Gram-positive bacteria, we incubated a selection of Gram-positive clinical isolates with the native LPETG substrate [28], [29]. *S. agalactiae*, *E. faecalis* and *S. cohnii* showed no incorporation of the native LPETG substrate. The VISA strain incorporated native LPETG substrate comparably to 8325-4 WT strain. Two out of the three tested *E. faecium* strains incorporated the native LPETG substrate, in particularly the *vanB* positive strain incorporated high levels of the SrtA specific substrate (Fig. 2B).

### The Incorporation of the Native LPETG Substrate in *S. aureus* Strains is Growth-phase Dependent and Peaks at the Stationary Growth Phase

**Figure 3:**
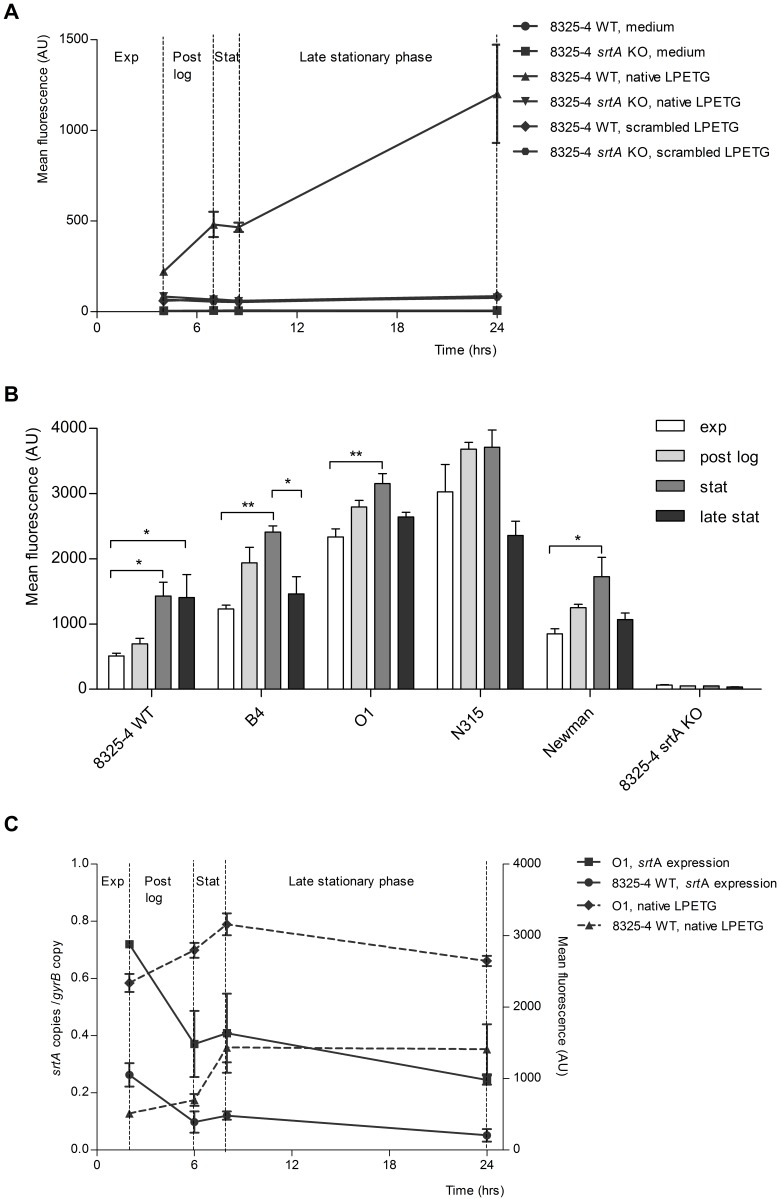
The SrtA-specific native LPETG substrate incorporation in 8325-4 WT bacteria occurs in a cumulative manner, is growth phase-dependent and negatively correlates to the srtA mRNA expression. (A) Native LPETG substrate incorporation during different bacterial growth phases. *S. aureus* 8325-4 WT and *srtA* KO were grown until different growth phases (exp: exponential, post log: post-logarithmic, stat: stationary or late stationary) in the presence of 1 mM native or scrambled LPETG substrates. Bacteria were harvested at several time points depicted on the x-axis. The mean fluorescence intensity is depicted on the y-axis. (B) Determination of the SrtA activity towards the native LPETG substrate in *S. aureus* bacteria in different bacterial growth phases. Five WT *S. aureus* strains and one *S. aureus srtA* KO strain were cultured in LB medium in the absence of the substrate until exponential, post-logarithmic, stationary or late stationary phase. Normalized bacteria to OD_600 nm_ 0.5 were incubated with the native or scrambled LPETG substrate in SrtA buffer during 1 hr. The bacteria were analysed with FACS after several PBS washes. Bacteria tested are depicted on the x-axis and the mean fluorescence intensity is depicted on the y-axis. We used one-way ANOVA testing with Bonferroni correction to analyse statistical differences. (C) Determination of the *srtA* expression during different growth phases. 8325-4 WT and O1 *S. aureus* strains were cultured until the previously mentioned growth phases at which mRNA was isolated. The time points at which the bacteria were harvested are plotted on the x-axis. The mean fluorescence reflecting the native LPETG substrate incorporation in the corresponding strains was plotted for the comparison on the right y-axis. The mRNA transcripts are depicted on the left y-axis. *srtA* transcripts were quantified and compared to the transcription of *gyr*B.

We wondered whether SrtA is capable to incorporate the native LPETG substrate during the different growth phases. We cultured 8325-4 WT *S. aureus* strain in the presence of the native or scrambled LPETG substrates until exponential, post-logarithmic, stationary or late stationary phase, and quantified the substrate incorporation using FACS. A time dependent progressive accumulation of native LPETG in the cell-wall was observed (r = 0.99), suggesting that in each growth phase SrtA is active (Fig. 3A). To explore the *S. aureus* SrtA activity in these growth phases in greater detail, different *S. aureus* strains were cultured in the absence of the substrate until they had reached a specific growth phase. The bacteria were harvested, and then incubated with native LPETG for one hour. Incorporation of native LPETG was subsequently measured by FACS. In all tested strains, the incorporation rate was lowest at the exponential phase, intermediate during the post-logarithmic phase, and in the majority of the strains peaked at the stationary phase (in 4 out of the 5 tested strains, the incorporation rate was two times higher in this phase than in the exponential phase), and decreased in the late stationary phase (Fig. 3B).

In order to determine whether the increased SrtA activity in stationary phase bacteria was due to the increased expression of sortase A, transcription of *srtA* was measured in two representative strains (8325-4 WT and O1) during the various bacterial growth phases. The overall *srt*A expression was higher in the clinical isolate (O1) compared to the laboratory strain 8325-4 WT. This is in line with the observation that O1 strain shows an increased incorporation of the native LPETG substrate in comparison to 8325-4 WT (Fig. 3C). In both strains s*rtA* mRNA expression peaked during the exponential growth phase and then decreased over time in a growth phase-dependent manner (Fig. 3C). Overall, *srtA* mRNA expression negatively correlated to the native LPETG substrate incorporation rate (r = −0.70 for 8325-4 WT strain and r = −0.54 for O1 strain).

### Native LPETG Substrate Incorporation in *S. aureus* 8325-4 WT Strain Does not Affect Incorporation of Physiological SrtA Substrates

**Figure 4:**
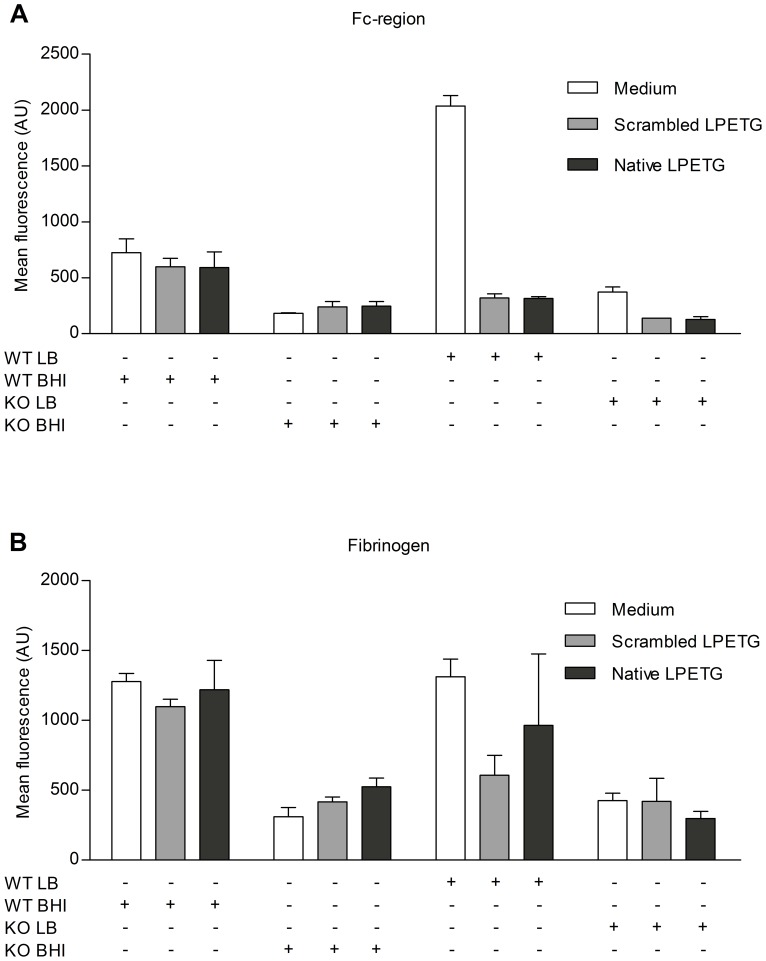
Fibrinogen and IgG Fc region binding to *S. aureus* bacteria is not specifically influenced by the native LPETG substrate incorporation. *S. aureus* 8325-4 WT and *srtA* KO strains were grown until exponential growth phase in LB and BHI medium in the presence of the native or scrambled LPETG substrates. After 3 PBS washes, the bacteria were incubated with fluorescently labelled (A) IgG Fc region or (B) fibrinogen. The IgG Fc region and fibrinogen binding was analysed with FACS. The conditions tested are depicted under the graphs. The mean fluorescence intensity is depicted on the y-axis.

**Figure 5:**
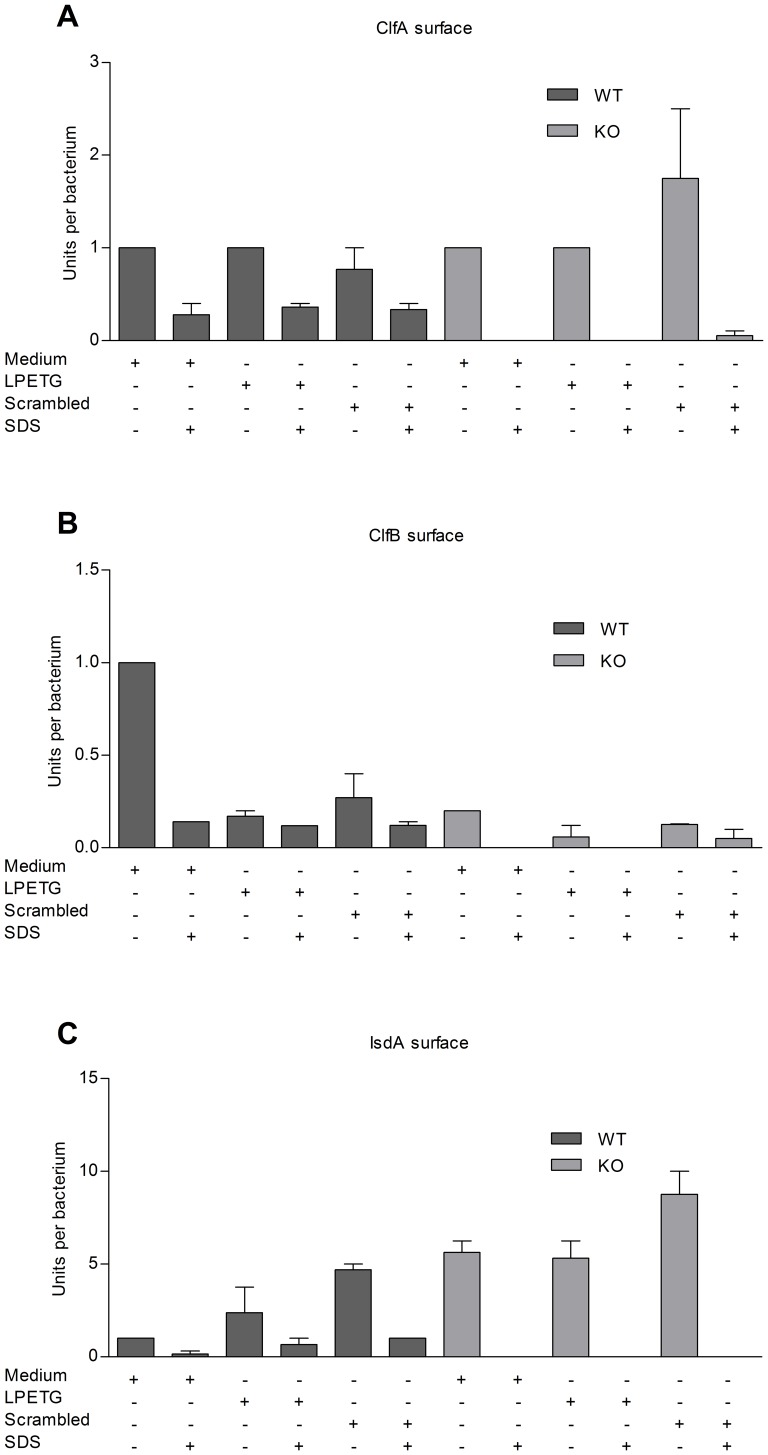
Native LPETG substrate incorporation does not lead to a decreased detection of ClfA, ClfB and IsdA cell-surfaces-associated proteins on the bacterial surface. 8325-4 WT and its isogenic *srtA* KO *S. aureus* strains were cultured until exponential growth phase in the presence of the native or scrambled LPETG substrate in BHI medium. The competition of the synthetic substrate with the physiological *S. aureus* substrates (A) ClfA, (B) ClfB and (C) IsdA was analysed with a competitive Luminex assay [25]. Based on the log dose-response curves of tested *S. aureus* cell-surface-associated proteins, a semi quantitative relative signal for the tested cell-surface-associated proteins was calculated by comparing the signals of the test samples with that of the control (normalized to 1 unit per bacterium). 8325-4 WT strain cultured only in medium was used as a control. The tested conditions are depicted under the graphs. The semi quantitative relative signals of the tested *S. aureus* physiological cell-surface-associated proteins are depicted on the y-axis as units per bacterium.

**Figure 6:**
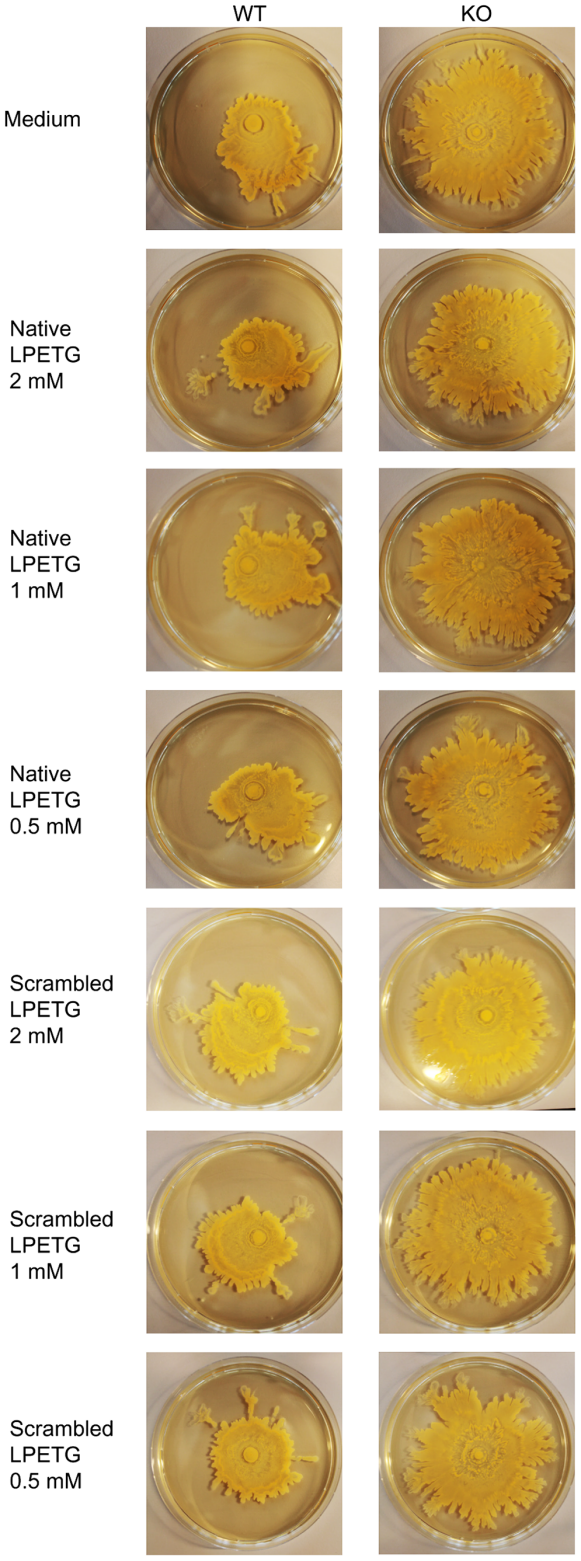
Native LPETG substrate incorporation does not induce the srtA KO hyper-spreading phenotype in the WT S. aureus strain. SH1000 WT and its isogenic *srtA* KO *S. aureus* strains were cultured in TSB in the presence of either medium, native or scrambled LPETG substrates during 24 hrs. The final substrate concentration was 2 mM, 1 mM or 0.5 mM. After the incubation individual cultures were spotted on the TSA soft agar and incubated during 20 hrs. The spreading zones were examined and pictures were taken after the incubation. These are representative images of three independent experiments.

To determine whether exogenous native LPETG substrate competes with the native SrtA-dependent cell-surface-associated proteins on the *S. aureus* 8325-4 WT surface, bacteria were grown in the presence of native LPETG in different media until exponential growth phase. Fibrinogen and Fc binding properties were subsequently measured with FACS. No specific alternations in the levels of fibrinogen and IgG Fc region binding to 8325-4 WT were observed, neither in BHI nor in LB medium (Fig. 4A–B). Furthermore, no decrease in surface exposition of the physiological SrtA substrates ClfA, ClfB and IsdA occurred, as measured by a competitive Luminex assay (Fig. 5A–C). Finally, we examined if culturing in the presence of native LPETG would induce a SrtA KO hyper-spreading phenotype. Culturing *S. aureus* strain in the presence of the native LPETG substrate, did not lead to the induction of such a phenotype (Fig. 6).

## Discussion

Nelson *et al* previously demonstrated that *S. aureus* endogenous SrtA is accessible for exogenous LPETG-containing substrate and, as such, is capable of incorporating this substrate covalently in a SrtA-dependent manner into the bacterial cell-wall [17]. We have confirmed these data with three independent *in vitro* experiments. The native LPETG substrate incorporation occurred despite the production of the native SrtA-dependent cell-surface-associated proteins. The native LPETG substrate incorporation occurred in the absence of the positively charged region and the hydrophobic part naturally occurring on the C-terminus of the SrtA-dependent cell-surface-associated proteins. Our data and the data of Nelson *et al*, therefore, do not support the data of Schneewind *et al* who found C-terminal hydrophobic domain and the positively charged tail of the transmembrane domain to be critical for the successful covalent incorporation of SrtA-dependent cell-surface-associated proteins into the peptidoglycan layer of living *S. aureus* bacteria [11]. This suggests that the C-terminal hydrophobic domain and the positively charged tail are only essential for endogenous cell-surface-associated proteins incorporation exported from the cytoplasm.

Besides *S. aureus*, a selection of Gram-positive clinical isolates incorporated the native LPETG substrate as well. This might be a consequence of the fact that SrtA transpeptidase is widely distributed among Gram-positive bacteria [29].

From our experiments we conclude that endogenous *S. aureus* SrtA incorporates most native LPETG substrate during stationary growth. We hypothesised that *S. aureus* may possess more available SrtA in the stationary phase due to competition of cell-surface-associated proteins during other growth phases, in particular the exponential phase, where the highest *srtA* expression occurs [30]. To support this hypothesis, we studied a *sarA* mutant. The *sar*A locus is responsible for the regulation of the production of many cell-surface-associated proteins [31], [32]. We found no difference in the ability of the *sarA* mutant to incorporate the native LPETG substrate in comparison to a 8325-4 WT strain. This finding suggests that the cell-surface-associated proteins are not the limiting factor for the native LPETG substrate incorporation during different bacterial growth phases.

We did not find any effect on the native cell-surface-associated proteins deposition upon native LPETG exposure. The native LPETG substrate can be incorporated into the bacterial cell-wall in large quantities, comparable to the native collagen binding protein [17]. We suggest, therefore, that there are enough accessible and free pentaglycines during all bacterial growth phases, in particular stationary growth phase, in the bacterial cell-wall for both the native LPETG substrate and cell-surface associated proteins to be incorporated [33], [34].

*S. aureus* bacteria lacking functional SrtA are attenuated in their virulence [20], [35]–[37]. Therefore, SrtA is an attractive target for anti-microbial therapy. In the past, several attempts were made to develop SrtA inhibitors [38]. However, most of these inhibitors identified previously lack specificity, have a low specific activity or possess non-suitable chemical characteristics such as high molecular weight and undesirable pharmacological properties, which render these inhibitors unsuitable as potential therapeutics. Alternatively, our synthetic SrtA specific substrates might be used as a vehicle for antibiotics or anti-microbial peptides to introduce more specific targeting of these molecules into the bacterial cell-wall. This specific targeting might in the future facilitate peptide-based therapy, which is an interesting alternative on conventional anti-bacterial treatment.
